# Rationale and design of a randomised trial of trientine in patients with hypertrophic cardiomyopathy

**DOI:** 10.1136/heartjnl-2022-322271

**Published:** 2023-05-03

**Authors:** John Farrant, Susanna Dodd, Carly Vaughan, Anna Reid, Matthias Schmitt, Clifford Garratt, Mohammed Akhtar, Masliza Mahmod, Stefan Neubauer, Robert M Cooper, Sanjay K Prasad, Anvesha Singh, Ladislav Valkovič, Betty Raman, Zakariye Ashkir, Dannii Clayton, Olatz Baroja, Beatriz Duran, Catherine Spowart, Emma Bedson, Josephine H Naish, Chris Harrington, Christopher A Miller, TEMPEST investigators, Christopher Miller

**Affiliations:** 1 BHF Manchester Centre for Heart and Lung Magnetic Resonance Research, Manchester University NHS Foundation Trust, Manchester, UK; 2 Division of Cardiovascular Sciences, School of Medical Sciences, Faculty of Biology, Medicine and Health, Manchester Academic Health Science Centre, University of Manchester, Manchester, UK; 3 Liverpool Clinical Trials Centre, University of Liverpool, Liverpool, UK; 4 Department of Health Data Sciences, Faculty of Health and Life Sciences, University of Liverpool, Liverpool, UK; 5 Division of Cardiovascular Medicine, University of Oxford, Oxford, UK; 6 NIHR Oxford Biomedical Research Centre, Oxford University Hospitals NHS Foundation Trust, Oxford, UK; 7 Institute of Cardiovascular Medicine and Science, Liverpool Heart and Chest Hospital, Liverpool, UK; 8 School of Sport and Exercise Sciences, Liverpool John Moores University, Liverpool, UK; 9 Cardiology, Royal Brompton and Harefield Hospitals, London, UK; 10 Department of Cardiovascular Sciences, University of Leicester and the NIHR Leicester Biomedical Research Centre, Glenfield Hospital, Leicester, UK; 11 Department of Imaging Methods, Institute of Measurement Science, Slovak Academy of Sciences, Bratislava, Slovakia; 12 SAS Trace Element Laboratory, Surrey Research Park, Guildford, UK; 13 Royal Surrey NHS Foundation Trust, Guildford, UK; 14 Wellcome Centre for Cell-Matrix Research, Division of Cell-Matrix Biology & Regenerative Medicine, School of Biology, Faculty of Biology, Medicine & Health, Manchester Academic Health Science Centre, University of Manchester, Oxford Road, Manchester, M13 9PT, UK

**Keywords:** hypertrophic cardiomyopathy, cardiomyopathy, hypertrophic, pharmacology, clinical

## Abstract

**Aims:**

Hypertrophic cardiomyopathy (HCM) is characterised by left ventricular hypertrophy (LVH), myocardial fibrosis, enhanced oxidative stress and energy depletion. Unbound/loosely bound tissue copper II ions are powerful catalysts of oxidative stress and inhibitors of antioxidants. Trientine is a highly selective copper II chelator. In preclinical and clinical studies in diabetes, trientine is associated with reduced LVH and fibrosis, and improved mitochondrial function and energy metabolism. Trientine was associated with improvements in cardiac structure and function in an open-label study in patients with HCM.

**Methods:**

The Efficacy and Mechanism of Trientine in Patients with Hypertrophic Cardiomyopathy (TEMPEST) trial is a multicentre, double-blind, parallel group, 1:1 randomised, placebo-controlled phase II trial designed to evaluate the efficacy and mechanism of action of trientine in patients with HCM. Patients with a diagnosis of HCM according to the European Society of Cardiology Guidelines and in New York Heart Association classes I–III are randomised to trientine or matching placebo for 52 weeks. Primary outcome is change in left ventricular (LV) mass indexed to body surface area, measured using cardiovascular magnetic resonance. Secondary efficacy objectives will determine whether trientine improves exercise capacity, reduces arrhythmia burden, reduces cardiomyocyte injury, improves LV and atrial function, and reduces LV outflow tract gradient. Mechanistic objectives will determine whether the effects are mediated by cellular or extracellular mass regression and improved myocardial energetics.

**Conclusion:**

TEMPEST will determine the efficacy and mechanism of action of trientine in patients with HCM.

**Trial registration numbers:**

NCT04706429 and ISRCTN57145331.

**WHAT IS ALREADY KNOWN ON THIS TOPIC**

- Therapies targeting underlying disease mechanisms in hypertrophic cardiomyopathy (HCM) remain limited. Trientine is a highly selective copper II chelator that is associated with reduced left ventricular (LV) hypertrophy and fibrosis, and improved mitochondrial function and energy metabolism, in diabetes. In a pilot study in HCM, trientine was associated improvements in cardiac structure and function.

**WHAT THIS STUDY ADDS**

- The Efficacy and Mechanism of Trientine in Patients with Hypertrophic Cardiomyopathy (TEMPEST) is a multicentre, double-blind, randomised, placebo-controlled phase II trial designed to evaluate the efficacy and mechanism of action of trientine in HCM.
- Primary outcome is change in LV mass indexed to body surface area. Secondary outcomes include exercise capacity and arrhythmia burden.
- Mechanistic objectives will determine whether the effects are mediated by cellular or extracellular mass regression and improved myocardial energetics.

**HOW THIS STUDY MIGHT AFFECT RESEARCH, PRACTICE OR POLICY**

- TEMPEST will determine the efficacy and mechanism of action of trientine in patients with HCM.

## Introduction

Hypertrophic cardiomyopathy (HCM), the most common inherited cardiac disorder, is a heart muscle disease most often caused by variants in one or more sarcomeric genes.^1 2^ Pathophysiologically, HCM is characterised by left ventricular (LV) hypertrophy, cardiomyocyte disarray and myocardial fibrosis.

Clinical manifestations are variable but can be profound. Two-thirds of patients have symptoms at diagnosis, including breathlessness, chest pain, effort intolerance, palpitations and syncope.^3^ One in 20 develop advanced heart failure within 5–8 years, and 1 in 25 have a stroke or peripheral embolism annually.^1 2 4 5^ Importantly, approximately 1% of patients die suddenly each year due to ventricular arrhythmia. Clinical manifestations are particularly deleterious given the young age of patients; mean age at diagnosis is around 40, and HCM has been reported to be the leading cause of sudden death in people aged under 35.^6^

Management traditionally comprises therapies to palliate symptoms and implantable cardioverter–defibrillators to prevent sudden death in patients deemed to be at high risk. More recently, trials of Mavacamten, an allosteric inhibitor of β-cardiac myosin ATPase activity, have demonstrated reduced LV outflow tract gradient and improved symptoms, exercise performance and health status in patients with symptomatic obstructive HCM (LV outflow tract gradient of 50 mm Hg or greater and New York Heart Association (NYHA) class II and III symptoms).^7 8^ There remains a ‘critical need’ for therapies that ‘can target pathways of HCM disease expression and, thereby, improve on the natural history of patients with this disease’.^9^

### Rationale for trientine in HCM

Trientine dihydrochloride is a highly selective copper II chelator that is licensed in Wilson disease, a genetic disorder of copper excretion. Patients with Wilson disease can exhibit a cardiac phenotype that mimics HCM.

Type II diabetes is associated with LV hypertrophy and abnormal copper homeostasis.^10^ In preclinical diabetic models, trientine is associated with reduced LV hypertrophy and fibrosis, and improved LV function, cardiomyocyte structure and organisation of muscle fibres.^11–15^ In a randomised placebo-controlled trial in patients with type 2 diabetes and LV hypertrophy, 12 months of trientine dihydrochloride 1200 mg/day was associated with a significant reduction in LV hypertrophy (change in left ventricular mass indexed to body surface area (LVMi) with trientine: −10.6±7.6 g/m^2^ vs placebo −0.1±9.8 g/m^2^; p<0.01) without changes in blood pressure or glucose.^16^ The decrease in LVMi was independently determined by the trientine-induced cumulative urinary copper excretion, and urine copper excretion was higher in patients with higher baseline LV mass. As such, trientine appears to modulate key pathological features of HCM, that is, LV hypertrophy, cardiomyocyte disarray and fibrosis.

HCM is associated with altered copper homeostasis, specifically, elevated serum copper and caeruloplasmin, in comparison to matched healthy volunteers.^17^ In an open-label pilot study of 20 patients with HCM, trientine dihydrochloride at a dose of 1200 mg/day for 6 months was associated with non-significant decreases LV mass (baseline: 152±54 g, follow-up: 147±55 g; p=0.06) and myocardial fibrosis, measured using cardiovascular magnetic resonance (CMR) extracellular volume (baseline: 30.0%±4.5%, follow-up: 29.5%±4.0%; p=0.06), and significant improvements in LV global longitudinal strain (baseline: −18.3%±3.4%, follow-up: −19.4%±3.4%; p=0.03) and total left atrial strain (baseline: 20.0%±3.9%, follow-up: 21.5%±5.0%; p=0.04).^18^ Trientine was associated with an increase in urine copper (baseline: 0.42±0.2 µmol/L/24 hours, follow-up: 2.02±1.0 µmol/L/24 hours; p=0.001) but no change in serum copper. The results were encouraging despite treatment duration being only 6 months, compared with 12 months in the trial in diabetes, in which LV mass reduction at 12 months was double that at 6 months.^16^

### Potential mechanism of action of trientine in HCM

Unbound/loosely bound tissue copper II ions are powerful catalysts of reactive oxygen species (ROS) and oxidative stress, and inhibitors of enzymatic antioxidants such as extracellular superoxide dismutase. Trientine has a range of actions thought to result from its removal of copper II ions from tissue. Trientine is associated with restoration of mitochondrial ultrastructure and normalisation of myocardial expression and enzymatic activity of proteins involved with energy metabolism, components of the mitochondrial respiratory chain and enzymes involved in fatty acid oxidation.^11 19^ This is potentially highly relevant to HCM because energy depletion is widely hypothesised to be a mechanism by which gene variants lead to the phenotype.^20^ Significantly impaired myocardial energetics (reduced phosphocreatine (PCr) to ATP ratio, measured using phosphorus-31 magnetic resonance spectroscopy (^31^P MRS)) are observed in HCM sarcomeric variant carriers before they develop LV hypertrophy, suggesting energy deficiency may be a primary event.^21^ Impaired myocardial energetics in HCM are also associated with progressive myocardial fibrosis.^22^ Furthermore, inherited defects in mitochondrial energy production and fatty-acid oxidation lead to phenotypes mimicking HCM.^23^ Trientine also normalises extracellular superoxide dismutase, which inhibits ROS-mediated tissue growth factor-β (TGF-β) activation and reverses myocardial fibrosis.^12 14^

## Trial design and methods

### Overall study design and governance

**Figure 1:**
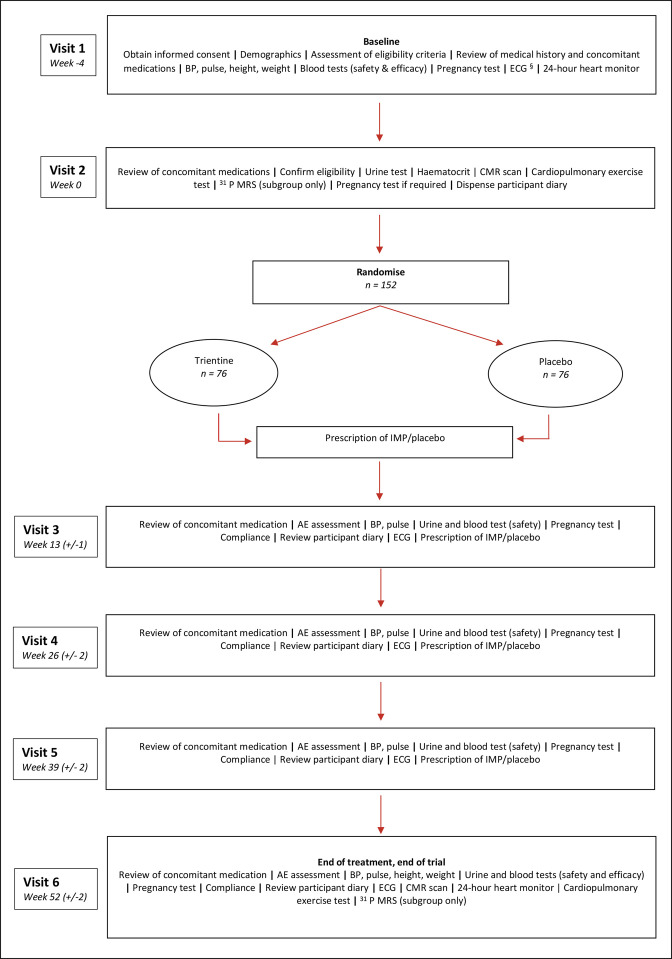
TEMPEST trial design. §May be performed at visit 1 or visit 2 but must be done before randomisation. AE, adverse event; BP, blood pressure; CMR, cardiovascular magnetic resonance; IMP, investigational medicinal product; ^31^P MRS, phosphorus-31 magnetic resonance spectroscopy; TEMPEST, The Efficacy and Mechanism of Trientine in Patients with Hypertrophic Cardiomyopathy.

The Efficacy and Mechanism of Trientine in Patients with Hypertrophic Cardiomyopathy (TEMPEST) is a multicentre, double-blind, parallel group, 1:1 randomised, placebo-controlled phase II clinical trial designed to evaluate the efficacy and mechanism of action of trientine in patients with HCM. The overall study design is summarised in figure 1. The efficacy hypothesis is that trientine will reduce LV mass, which will be associated with improved exercise capacity, reduced arrhythmia burden and improved cardiac function. The mechanistic hypothesis is that the reduction in LV mass will be mediated by a reduction in myocardial cellular mass and fibrosis and improved myocardial energetics, which will be determined by increased copper excretion. The trial was designed by the research team with patient and public involvement and has been registered.

Box 1 **Eligibility criteria for TEMPEST**

**Inclusion criteria**

- Written informed consent.
- Age 18–75 inclusive.*
- HCM, as defined by the ESC HCM guidelines as ‘a wall thickness≥15 mm in one or more LV myocardial segments that is not explained solely by loading conditions’^1^ The same definition is applied to first-degree relatives of patients with HCM; that is, all participants are required to have a LV wall thickness of ≥15 mm. Wall thickness is as measured on the most recent CMR scan performed prior to the baseline visit. If CMR has not been performed previously, wall thickness measurement should be taken from the most recent echocardiogram performed prior to the baseline visit.
  (It is recognised that in the ESC guidelines, a clinical diagnosis of HCM in first-degree relatives requires a wall thickness that is less than this value; however, ≥15 mm is applied here in order to ensure that all participants have an unequivocal phenotype).
- NYHA class I, II or III at the most recent clinical assessment performed prior to the baseline visit.

**Exclusion criteria**

- Previous or planned septal reduction therapy.
- Previously documented myocardial infarction or severe coronary artery disease.
- Uncontrolled hypertension, defined as a systolic blood pressure of >180 mm Hg or a diastolic blood pressure of >100 mm Hg at visit 1.
- Known LV EF of <50%, as measured on the most recent CMR scan performed prior to the baseline visit. If CMR has not been performed previously, the most recent echocardiogram performed prior to the baseline visit should be used.
- Previously documented persistent atrial fibrillation.
- Anaemia, defined as haemoglobin being below the local site normal reference range, at visit 1.
- Iron deficiency, defined as serum iron being below the local site normal reference range, at visit 1.
- Copper deficiency, defined as serum copper being below the normal reference range, at visit 1.
- Pacemaker or implantable cardioverter defibrillator.
- Known severe valvular heart disease, as demonstrated on the most recent heart imaging performed prior to the baseline visit.
- Previously documented other cardiomyopathic cause of myocardial hypertrophy (eg, amyloidosis, Fabry disease and mitochondrial disease).
- History of hypersensitivity to any of the components of the investigational medicinal product.
- Known contraindication to MRI scanning.
- Pregnancy, lactation or planning pregnancy. Women of childbearing capacity are required to have a negative serum pregnancy test before treatment, must agree to pregnancy tests at study visits as defined in the protocol and must agree to maintain highly effective contraception as defined in the protocol.
- Any medical condition, which in the opinion of the investigator, may place the patient at higher risk from his/her participation in the study, or is likely to prevent the patient from complying with the requirements of the study or completing the study.

*The original upper age limit was 70 years, but this was increased to 75 years in a protocol amendment in order to aid recruitment. See online supplemental table 1 for further details.CMR, cardiovascular magnetic resonance; EF, ejection fraction; ESC, European Society of Cardiology; HCM, hypertrophic cardiomyopathy; LV, left ventricular; NYHA, New York Heart Association; TEMPEST, The Efficacy and Mechanism of Trientine in Patients with Hypertrophic Cardiomyopathy.

10.1136/heartjnl-2022-322271.supp1 Supplementary data

**Table 1:** Visit schedule

Visit		1	2	3	4	5	6	
Time and acceptable window (weeks)		-4	0*	13±1	26±2	39±2	52±2	
Procedures		Screening and consent	Randomisation				End of treatment, end of trial	Unscheduled visit
Signed consent form		X						
Demographics		X						
Review of medical history		X						
Assessment of eligibility criteria		X	X					
Review of concomitant medications		X	X	X	X	X	X	X
Pulse and blood pressure		X		X	X	X	X	X†
Height and weight		X					X	X†
Give urine sample bottle to participant		X	X	X	X	X		X†
Clinical laboratory	Urine test‡		X	X	X	X	X	
	Pregnancy test§	X	X¶	X	X	X	X	X†
	Blood tests (safety)**	X		X	X	X	X	X†
	High-sensitivity troponin blood test	X					X	
	Haematocrit blood test††		X					
	Sample handling/processing for central analysis	X	X	X	X	X	X	X†
	Specimen dispatch by post/courier	X	X	X	X	X	X	X†
Special procedure	ECG	X‡‡		X	X	X	X	X†
	CMR scan		X				X	
	24-hour heart monitor	X					X	
	Cardiopulmonary exercise test		X				X	
	Phosphorus spectroscopy (subgroup)		X				X	
Randomisation			X					
Prescription of IMP			X	X	X	X		
Pharmacy dispensing of IMP			X	X	X	X		
Dispense participant diary and instruct			X					
Pharmacy collection and recording of unused medication to assess compliance				X	X	X	X	X†
Review patient diary				X	X	X	X	X†
Review/reporting of AEs/SAEs				X	X	X	X	X
eCRF completion including data transfer and query resolution		X	X	X	X	X	X	X

Other abbreviations as per box 1.

*Visit 2 (randomisation) should take place within 28 days (4 weeks) of visit 1.

†Optional procedures performed at the investigator’s discretion

‡Urine test (efficacy): early morning urine collection for urine copper.

§Applies to females of childbearing capacity only. A serum pregnancy test will be performed at visit 1. Urine pregnancy tests will be performed at subsequent visits (serum testing may be performed if timely urine testing is not available).

¶At visit 2, female participants of childbearing capacity will be asked if there is a chance that they could have become pregnant since visit 1. If the participant confirms that there is a chance, then a urine pregnancy test will be performed.

**Blood tests (safety): blood count, renal function, serum iron, serum copper and serum caeruloplasmin.

††Haematocrit blood test (used as part of the CMR measurements)

‡‡May be performed at visit 1 or visit 2 but must be done before randomisation.

AE, adverse event; CMR, cardiovascular magnetic resonance; eCRF, electronic case report form; IMP, investigational medicinal product; SAE, serious adverse event.

**Table 2:** TEMPEST trial objectives and outcome measures

**Overall objective** To evaluate the clinical efficacy and mechanism of action of trientine in hypertrophic cardiomyopathy	
**Objective**	**Outcome measure**
Primary efficacy objective	
To determine whether trientine compared with placebo leads to regression of LV hypertrophy	Change in in LV mass indexed to body surface area
Secondary efficacy objectives To test whether trientine compared with placebo:	
Increases urinary copper excretion.	Cumulative urine copper excretion, measured using urinary copper
Improves exercise capacity.	Change in exercise capacity, measured using cardiopulmonary exercise testing
Reduces arrhythmia burden.	Change in number of non-sinus supraventricular heart beats, presence and amount of atrial fibrillation, number of ventricular-origin beats and presence and amount of non-sustained ventricular tachycardia, in 24 hours, measured using ambulatory heart monitoring
Reduces cardiomyocyte injury.	Change in circulating high sensitivity troponin.
Improves LV contractile function.	Change in LV global longitudinal strain and strain rate, wall thickness, mass, volumes and ejection fraction measured using CMR.
Improves left atrial structure and function.	Change in atrial volume and function, measured using CMR.
Mechanistic objectives To understand how trientine may cause a reduction in LV hypertrophy the study will determine whether:	
Trientine, compared with placebo, leads to cellular or extracellular mass regression.	Change in LV myocardial cellular mass, myocardial extracellular mass, myocardial extracellular volume, LV late gadolinium enhancement, measured using CMR
Trientine, compared with placebo, leads to an improvement in myocardial energetics.	Change in PCr:ATP ratio, measured using ^31^P MRS (subroup)
LV hypertrophy regression is mediated by cellular regression, extracellular regression or improved myocardial energetics, which are in turn determined by copper excretion.	Mediation analysis, using the aforementioned outcome measurements.

CMR, cardiovascular magnetic resonance; LV, left ventricular; PCr:ATP, phosphocreatine to ATP; ^31^P MRS, phosphorus-31 magnetic resonance spectroscopy.

TEMPEST is conducted by the research team, in conjunction with Liverpool Clinical Trials Centre, a UK Clinical Research Collaboration fully registered clinical trials unit. The sponsor is Manchester University NHS Foundation Trust. The trial is funded by the Efficacy and Mechanism Evaluation Programme, a Medical Research Council and UK National Institute for Health and Care Research (NIHR) partnership (project reference NIHR127575). The funder had no role in trial design other than through their external peer review processes, and were not involved in the preparation, drafting or editing of this article. Univar Solutions B.V. has gifted the investigational medicinal product (IMP). Univar Solutions B.V. have had no role in trial design, and were not involved in the preparation, drafting or editing of this article. Univar Solutions B.V. conducted a factual accuracy check of this article, but any decisions to incorporate comments were made solely at the discretion of the authors. All authors reviewed and approved the manuscript and assume full responsibility for its accuracy.

A Trial Steering Committee provides overall supervision for the trial and provides advice through its independent Chairman. An independent data and safety monitoring committee is responsible for reviewing and assessing recruitment, interim monitoring of safety and effectiveness, trial conduct and external data, and submits periodic reports to the Trial Steering Committee. Further details are available in the online supplemental material.

### Patients

The eligibility criteria are summarised in box 1. Briefly, patients are 18–75 years of age, with a diagnosis of HCM in keeping with the European Society of Cardiology HCM guidelines, that is, ‘a wall thickness≥15 mm in one or more LV myocardial segments that is not explained solely by loading conditions’,^1^ and NYHA class I, II or III. Key exclusion criteria include previous or planned septal reduction therapy, uncontrolled hypertension, known LV ejection fraction of <50%, previously documented persistent atrial fibrillation, previously documented other cardiomyopathic cause of myocardial hypertrophy (eg, amyloidosis, Fabry disease or mitochondrial disease), anaemia, iron deficiency, copper deficiency, pacemaker or implantable cardioverter–defibrillator and contraindication to MRI scanning.

### Study procedures

#### Baseline evaluations

Potential participants are identified at NHS hospital trusts in the UK and invited to a baseline visit. After informed consent, participants undergo assessment of eligibility criteria, review of medical history and concomitant medications, measurement of vital signs, biochemistry and haematological laboratory investigations, ECG and 24-hour heart monitoring. If eligibility is confirmed, visit 2 is arranged during which a urine sample is collected and participants undergo CMR scanning and cardiopulmonary exercise testing before being randomised.

#### Randomisation

Participants are randomised in a 1:1 ratio to receive either trientine or placebo. Randomisation is accomplished over the internet using web randomisation software accessed using a secure website provided by the clinical trials unit. Block randomisation, stratified by site, is implemented, with computer generated randomisation allocations and randomly varying block sizes. The randomisation code has been generated by an independent clinical trials unit statistician who is not involved with TEMPEST.

#### Study visits and monitoring

Following randomisation, study visits occur at or around weeks 13, 26, 39 and 52. Unscheduled visits can also be performed at the discretion of the investigator. The visit schedule is shown in table 1. At follow-up visits, participants undergo a review of concomitant medications, assessment for adverse events and compliance, measurement of vital signs, biochemistry and haematological laboratory investigations and an ECG. At the final visit (week 52), baseline procedures are repeated to assess the primary and secondary outcome measures.

### Investigational medicinal product

The active treatment is trientine, taken orally as two Cufence 200 mg hard capsules two times per day (total daily dose of trientine is 800 mg, which is equivalent to 1200 mg of trientine dihydrochloride). The comparator is placebo, manufactured to appear identical to Cufence 200 mg hard capsules, taken orally as two capsules two times per day. The treatment period is 52 weeks. IMP dose may be reduced if participants experience adverse events, with subsequent re-escalation as appropriate. The IMP is taken in addition to participants’ clinical medication regimen, which is recorded at each visit.

### Subgroup

A subgroup of participants is undergoing ^31^P MRS, for measurement of myocardial energetics (PCr:ATP ratio), alongside the CMR at visit 2 and at the final visit.

### Protocol amendments

Modifications to the TEMPEST protocol are summarised in online supplemental table 1.

### Study outcomes

The primary objective is to determine whether trientine compared with placebo leads to regression of LV hypertrophy. The primary outcome measure is change in LVMi (g/m^2^), measured using CMR, from baseline to week 52. The secondary efficacy objectives are to determine whether trientine compared with placebo increases urinary copper excretion, improves exercise capacity, reduces arrhythmia burden, reduces cardiomyocyte injury, improves LV contractile function, reduces LV outflow tract gradient, and improves left atrial structure and function. The mechanistic objectives, which aim to understand how trientine may cause a reduction in LV hypertrophy, are to determine whether trientine compared with placebo leads to cellular or extracellular LV mass regression and an improvement in myocardial energetics, and to determine whether LV hypertrophy regression is mediated by myocardial cellular regression, extracellular regression or improved myocardial energetics, and, in turn, whether these are determined by urinary copper excretion. Corresponding outcome measures are described in table 2.

The trial is also evaluating the safety of trientine in HCM and whether treatment effect varies according to genotype. Screening and recruitment data and clinical endpoints are recorded to inform a subsequent phase III trial. Consent is requested to enable long-term follow-up using routinely collected healthcare data with appropriate linkage.

### Statistical considerations

#### Sample size

In the pilot study, the SD of within-patient differences in LVMi from baseline in the trientine group was 4.5 g/m^2^ and the SD in the observational control group was 2.4 g/m^2^. Using a conservative SD of within-patient differences from baseline of 5 g/m^2^ in both groups, we found that 64 patients per group are required to detect a minimum difference between the trientine and placebo groups of 2.5 g/m^2^ in terms of change LVMi from baseline following 52 weeks of treatment (80% power, 5% significance level, two-sided). To allow for treatment discontinuation in 25%, this was originally inflated to 86 per group (ie, total study n=172). However, trial retention was found to be better than expected; therefore, the study protocol was modified to reduce the treatment discontinuation rate to 15%, meaning that 76 patients per group are required (ie, total study n=152).

Sample size calculations for the mechanistic outcomes are given in the online supplemental material.

#### Statistical analyses

The trial will be analysed and reported using the Consolidated Standard of Reporting Trials and the ICH E9 guidelines. A full and detailed statistical analysis plan has been developed, the main features of which are described briefly here. Data management and statistical analysis are performed independently by the clinical trials unit.

Primary analysis will be by intention to treat, using complete case analysis, with a sensitivity regression analysis for the primary outcome (adjusting for variables which predict outcome to account for missingness).^24^ LVMi (and other outcome measures) will be compared between groups using analyses of covariance, adjusting for baseline values. Correlation analysis will assess relationships between outcome parameters. A conventional 5% significance level will be used.

An additional sensitivity analysis will estimate causal effect of treatment on the primary outcome by appropriately allowing for dose received using instrumental variable regression, thus accounting for informative premature treatment discontinuation.

Potential mediators of treatment on LVMi include myocardial fibrosis (extracellular mass), cellular mass, PCr:ATP ratio and copper excretion. In order to test whether these variables predict change in LVMi, mediation analysis will be carried out, adjusting for baseline covariates that predict both the mediator and LVMi. Sensitivity analyses will be conducted to assess the potential impact of unmeasured confounding between the mediator and outcome.

## Discussion

Therapies that target the underlying disease pathways in HCM remain limited. Selective copper II chelation with trientine represents a novel approach, supported by considerable preclinical and clinical data.

Copper is an essential trace element, with roles in multiple biological processes, including signalling pathways, cellular respiration and as an enzymatic cofactor. It is present in humans in reduced (copper I or Cu^+^) and oxidised (copper II or Cu^2+^) forms.^25^ Unbound/loosely bound tissue copper II ions are powerful catalysts of ROS and oxidative stress and inhibitors of enzymatic antioxidants such as extracellular superoxide dismutase.^26^ ROS and enhanced oxidative stress damage mitochondrial structure and function, interrupting energy metabolism, activate TGF-β and other profibrotic mediators leading to myocardial fibrosis, and stimulate cardiomyocyte hypertrophy. HCM is characterised by LV hypertrophy and myocardial fibrosis, with enhanced oxidative stress and energy depletion representing key pathophysiological mechanisms.^27^

The beneficial cardiac effects of trientine observed preclinically and clinically in type 2 diabetes include reduced LV hypertrophy and fibrosis, and improved LV function, cardiomyocyte structure and organisation of muscle fibres. These benefits are thought to result from the removal of unbound/loosely bound copper II ions from myocardium, which attenuates oxidative stress.^11 12^ Trientine restores mitochondrial ultrastructure and normalises myocardial expression and enzymatic activity of proteins involved with energy metabolism, components of the mitochondrial respiratory chain and enzymes involved in fatty acid oxidation.^19^ It normalises extracellular superoxide dismutase, which inhibits ROS-mediated TGF-β activation. Consistent with these salutary effects, the encouraging results from the open-label pilot study of trientine in patients with HCM provide strong rationale for the TEMPEST trial.^18^

In one of the largest randomised placebo-controlled trials in HCM, TEMPEST will provide comprehensive evaluation of the clinical efficacy and mechanism of action of trientine in HCM. In contrast to some contemporary trials in HCM, eligibility criteria are broad, and thus the findings will be applicable to almost all patients with HCM (online supplemental table 2).

Although the choice of primary outcome for trials in HCM is challenging, LV hypertrophy is the defining feature of HCM, while also independently predictive of adverse outcome and a key determinant of effort intolerance.^28 29^ LVMi was therefore appropriately selected as the primary outcome, and measurement with CMR ensures high reproducibility. Importantly, TEMPEST also includes assessment of exercise capacity with cardiopulmonary exercise testing, heart rhythm and other aspects of cardiac structure and function. The detailed evaluation of mechanism of action, including assessment of myocardial fibrosis, cellular mass and energy metabolism, coupled with the mediation analysis, adds further strength to the trial. Myocardial fibrosis is an important determinant of outcome in HCM in its own right, and measurement of myocardial energetics provides nuanced mechanistic insight.

The rationale for the selected dose of trientine is that it is the dose used in the aforementioned studies in diabetes and HCM, in which it was associated with the described beneficial/potentially beneficial effects, and was safe and well tolerated.^16 18^ A duration of 52 weeks was chosen because it was felt to be the shortest period within which efficacy can be demonstrated while also balancing value for money and minimising patient burden. As described, LV mass reduction at 12 months was double that at 6 months in the trial in type 2 diabetes.^16^

In summary, TEMPEST will determine the efficacy and mechanism of action of trientine in patients with HCM.

## Data Availability

No data are available.
