# Supplementary material for: Rationale and design of a randomised trial of trientine in patients with hypertrophic cardiomyopathy
Source: Heart. 2023 May 3;109(15):1175–82. doi: 10.1136/heartjnl-2022-322271 (PMC10359575; doi:10.1136/heartjnl-2022-322271)
Supplement: Supplementary data [file heartjnl-2022-322271supp001.pdf]

Trientine in hypertrophic cardiomyopathy: rationale and design of the TEMPEST trial

Supplementary material

Farrant J, *et al. Heart* 2023; 109:1175–1182. doi: 10.1136/heartjnl-2022-322271

Oversight committees

Trial Steering Committee

Colin Berry (Chair)

Catriona Graham

Saidi Mohiddin

Scot Garg

Andrew Auty (patient representative).

Independent Data Safety and Monitoring Committee

Sven Plein (Chair)

Marc Dweck

Nicola Greenlaw

Clinical trials unit

The trial is being conducted in conjunction with Liverpool Clinical Trials Centre, a UK Clinical Research Collaboration fully registered Clinical Trials Unit (UKCRC; www.ukcrc.org). The trials unit are responsible for trial management and monitoring, regulatory approvals, independent data management, randomisation and statistical analysis.

Farrant J, *et al. Heart* 2023; 109:1175–1182. doi: 10.1136/heartjnl-2022-322271

Trial procedures

Pulse, blood pressure, height and weight

Pulse

Pulse should be measured by direct palpation of usually the radial or brachial artery for 1 minute.

Blood pressure

Blood pressure should be measured using an automated or manual sphygmomanometer that has been appropriately calibrated locally. Either arm can be used.

Height

Height should be measured in centimetres to the nearest whole number. Shoes should not be worn.

Weight

Body weight should be measured in kilograms to the nearest 0.1 kilogram, in indoor clothing without shoes.

Blood and urine testing

Haemoglobin, haematocrit, white cell count, platelet count, eGFR and serum iron

Samples are sent to the local site laboratory for analysis.

Serum copper, serum caeruloplasmin and urine copper

Samples are analysed centrally at the SAS Trace Element Laboratory (Surrey Research Park, Guildford, Surrey, UK).

High sensitivity troponin

Samples are analysed centrally at Manchester University NHS Foundation Trust.

ECG

Farrant J, *et al. Heart* 2023; 109:1175–1182. doi: 10.1136/heartjnl-2022-322271

A standard 12-lead ECG is performed in line with local procedures. Measurements include sinus rhythm (Yes/ No), atrial fibrillation (Yes/ No), PR interval, QRS duration, QTc Interval.

Cardiovascular magnetic resonance (CMR)

CMR should be performed at 3T using a Siemens scanner, and before cardiopulmonary exercise testing. Participants should be scanned on the same scanner at baseline (visit 2) and at the end of the trial (visit 6). Participants with an estimated glomerular filtration rate < 30ml/min are not excluded from the trial, but they must not receive gadolinium-based contrast agent as part of their CMR scan. Breath-held acquisitions should be performed in expiration where possible. Dotarem (gadoteric acid) at a dose of 0.15 mmol/kg is the preferred gadolinium-based contrast agent.

Prior to scanning, standard safety checks should be performed in line with local procedures. A blood sample should be drawn and sent to the local site laboratory for measurement of haematocrit.

<u>Protocol</u>

1. Orthogonal localiser to adjust the heart to isocentre.
2. Axial bright-blood TrueFISP localiser: 20 or more single-shot axial slices to cover the thorax from just above aortic arch to just below left ventricular apex. Acquisition generally split over 2-3 breath-holds.
3. Localisers to derive the cardiac planes as per local procedure e.g., 2-chamber localiser, 4-chamber localiser, short-axis localiser.
4. 4-chamber steady state free-precession (SSFP) breath-hold cine. Ideally retrospective gating.
5. 2-chamber SSFP breath-hold cine. Ideally retrospective gating.
6. 3-chamber SSFP breath-hold cine. Ideally retrospective gating.
   (The order in which the 4-, 2-, and 3-chamber cines are acquired can be adjusted in line with local preference).
7. LVOT long-axis (i.e. perpendicular to the 3-chamber cine) SSFP breath-hold cine. Ideally retrospective gating.

Farrant J, *et al. Heart* 2023; 109:1175–1182. doi: 10.1136/heartjnl-2022-322271

8. Ventricular short-axis SSFP breath-hold cine stack. Ideally retrospective gating.

   8mm slices. No inter-slice gap.

   Check each slice for artefact and repeat as necessary.

   Check to ensure the base and apex of the left ventricle are fully covered.

9. Basal, mid and apical -ventricular short-axis T1 mapping.

   Basal slice position: Copy to the position of the most basal short-axis cine slice that does not contain any LVOT at end-diastole i.e. there should be a ‘complete ring’ of myocardium visible.

   Mid slice position: Copy to the position of the short-axis cine slice that is two slices more apical than the short-axis slice chosen as the basal slice.

   Apical slice position. Copy to the position of the short-axis cine slice that is two slices more apical than the short-axis slice chosen as the mid slice.

   Sequence: A MOdified Look-Locker Inversion Recovery (MOLLI) sequence should ideally be used. Breath-hold. Participants should undergo the same T1 mapping sequence at baseline (visit 2) and at the end of the trial (visit 6).

10. 3-chamber phase-encoded velocity mapping.

    Copy to the position of the 3-chamber SSFP cine.

    Set the velocity encoding (VENC) to 2.0 m/s.

    Free-breathing.

11. LVOT short-axis phase-encoded velocity mapping.

    Position the slice perpendicular to the LVOT using the 3-chamber and LVOT long-axis SSFP cines as reference.

    Use the 3-chamber phase-encoded velocity mapping to position the slice at the site of the apparent highest LVOT blood flow velocity.

    Set an appropriate VENC i.e. the lowest possible where aliasing is not expected to occur. A VENC of 2.0 m/s is the minimum that should be used, but, if an elevated LVOT blood flow velocity is expected based on the appearances of the cines, a higher VENC should be used e.g. 3.0 m/s. If aliasing occurs at the chosen VENC, the acquisition should be repeated using a VENC that is at least 1.0 m/s higher than that at which the aliasing occurred.

    Free-breathing.

12. Gadolinium-based contrast agent administration.

    The method of injection (power injector or by hand) should be as per local policy.

    A single scout left ventricular short-axis image should be acquired immediately following contrast injection and should be labelled as "Contrast injection end". (This will allow verification of the timing of injection).

13. Atrial short-axis SSFP breath-hold cine stack. Ideally retrospective gating.

    6mm slices. No inter-slice gap.

    Check each slice for artefact and repeat as necessary.

14. Inversion time scout.

    Acquire 6 minutes after gadolinium-based contrast agent administration.

    Copy to the position of the 4-chamber SSFP cine or a short-axis SSFP cine.

15. Ventricular short-axis late gadolinium enhancement imaging.

    Copy to the positions of the ventricular short-axis SSFP cine slices (so that the late gadolinium enhancement and cine images are directly comparable).

    8mm slices. No inter-slice gap.

    Sequence: Breath-hold segmented gradient echo phase sensitive inversion recovery, motion-correct if available.

    Start with the optimal inversion time as per the inversion time scout, and subsequently adjust as necessary.

16. Basal, mid and apical-ventricular short-axis T1 mapping.

    Repeat the basal and mid-ventricular short-axis T1 mapping using the same slice positions and sequence as were used before contrast agent administration.

    Begin the acquisition 15 minutes after contrast agent administration (start the timer when the contrast agent syringe is empty).

<u>Analysis</u>

CMR analysis is performed in a Central Core Lab at the BHF Manchester Centre for Heart and Lung Magnetic Resonance Research at Manchester University NHS Foundation Trust. Analysis is

performed using cvi42 (version 5.12.1; Circle Cardiovascular Imaging; Calgary, AB, Canada). Measurements are indexed to body surface area where appropriate.

Left ventricular mass, volumes and function, atrial volumes and function

LV mass, volumes, ejection fraction and wall thickness, and left atrial volumes are measured from SSFP images in accordance with recommendations from the Society for Cardiovascular Magnetic Resonance.[1] Papillary muscles and trabecular tissue are included with the myocardium as part of the measurement of LV mass. LV global longitudinal strain, strain rate and left atrial strain are measured using feature tracking as described previously.[2,3]

Native myocardial T1, extracellular volume and mass, and cellular mass

Measurements are made in accordance with recommendations from the Society for Cardiovascular Magnetic Resonance.[4] The middle third of myocardium on the T1 maps is used to measure myocardial T1. Extracellular volume (ECV) is calculated as: $ECV\ (\%) = \lambda\,(1 - haematocrit)$; where $\lambda = [\Delta R1_{myocardium}]/\ [\Delta R1_{bloodpool}]$ pre- and post-gadolinium contrast agent administration (where $R1 = 1/T1$). Myocardial extracellular mass is calculated as the product of LV mass and extracellular volume. Myocardial cellular mass is calculated as the product of LV mass and (1 – extracellular volume).

Late gadolinium enhancement (LGE)

Myocardial LGE is measured from short-axis LGE images using a signal intensity threshold of 5 standard deviations above a reference myocardial region without visible LGE.[1]

Peak LVOT gradient

Measured from the LVOT short-axis through-plane phase-encoded velocity map in accordance with recommendations from the Society for Cardiovascular Magnetic Resonance.[1]

Cardiopulmonary exercise test (CPET)

Farrant J, *et al. Heart* 2023; 109:1175–1182. doi: 10.1136/heartjnl-2022-322271

Sites can use either bike or treadmill exercise, but individual sites should use the same mode of exercise (bike or treadmill), and the same make and model of equipment, for all participants at their site. A maximal CPET is to be defined as that with a respiratory exchange ratio (RER) of >1.10. Prior to starting the CPET, usual safety checks should be performed in line with local procedures.

Bike protocol

1. Participants should have standard spirometry prior to the exercise portion of the study. This should include forced expiratory volume in 1 second (FEV1), forced vital capacity (FVC) and flow volume loop analysis.
2. Participants should be set up on the bike ensuring the correct ride height and handlebar position.
3. Participants should have continuous ECG monitoring applied.
4. Participants should have their mask fitted and checked for leaks prior to commencement of the test.
5. Breath by breath gas exchange analysis and continuous ECG monitoring must be performed throughout the test. Heart rate, peak heart rate and percentage predicted heart should be calculated. VCO_2 (litres/min), work rate (watts), heart rate and respiratory exchange ratio (RER) should be averaged every 10 seconds.
6. BP should be recorded at rest, warm up and every 3 minutes during the exercise phase and into the recovery phase until the BP has normalised and the participant has recovered.
7. Rest and warm up phases should last for 3 minutes.
8. A 15W Ramp protocol should be used.
9. Participants should be encouraged to continue the exercise phase for as long as possible aiming for an RER of >1.10.
10. Sites should record the reason for cessation of the exercise phase e.g. breathlessness, leg pain, chest pain.

Treadmill protocol

Farrant J, *et al. Heart* 2023; 109:1175–1182. doi: 10.1136/heartjnl-2022-322271

The treadmill protocol is the same as the bike protocol, except the modified Bruce protocol should be used.

Analysis

The CPET report generated is transferred to the Central Core Lab at the BHF Manchester Centre for Heart and Lung Magnetic Resonance Research at Manchester University NHS Foundation Trust. Exercise time, peak VO_2, anaerobic threshold and VE/CO_2 slope are recorded.

24-hour heart monitoring

Heart rhythm monitoring is performed using LifeSignals single-use patches. Following appropriate skin preparation, patches are be applied to the participant on the left side of the chest between the sternum and the left mid-clavicular line at the level of the second/third intercostal space.

Participants are instructed to wear the monitor for 24 hours, with instructions to avoid water going directly onto the patch. After 24 hours, participants should remove the monitor and send it in a pre-paid, pre-addressed envelope to the Central Core Lab at the BHF Manchester Centre for Heart and Lung Magnetic Resonance Research at Manchester University NHS Foundation Trust, where the recording is analysed using HE/LX analysis software (version 6.0c, Northeast Monitoring Inc). Measurements include number of non-sinus supraventricular heart beats per 1000 beats, paroxysmal atrial fibrillation (Yes/No), number of episodes of atrial fibrillation, total number of heart-beats in atrial fibrillation, number of ventricular-origin heart beats per 1000 beats, non-sustained ventricular tachycardia (Yes/No), number of episodes of non-sustained ventricular tachycardia, total number of heart-beats in non-sustained ventricular tachycardia.

Phosphorous magnetic resonance spectroscopy (^{31}P-MRS)

^{31}P-MRS is performed as part of the subgroup (Manchester and Oxford sites only). Participants are scanned supine, with a dual tuned radio-frequency coil placed over the heart. The position of the coil in respect to the heart is checked by acquiring a set of coronal localizers and identifying a set of cod liver oil capsules attached to the coil housing. To determine the loading of the coil, a set of

inversion recovery scans is acquired at a frequency of a phenyl phosphonic acid fiducial, which is also attached to the coil. The excitation frequency is set to -250 Hz from phosphocreatine (PCr), i.e. between γ- and α- adenosine triphosphate (ATP), to cover whole ^{31}P spectrum uniformly.

Acquisition

The Manchester and Oxford sites have different coils; thus, acquisition techniques are adjusted accordingly:

At the Manchester site, a 27 x 28 cm^2 transmit loop with a quadrature pair for receive coil is used. A 3-dimensional acquisition-weighted chemical shift imaging technique (3D UTE-CSI) is used with an acquisition matrix of 16 x 8 x 8 over field of view of 240 x 240 x 200 mm^3 with 10 averages at the centre of k-space. The chemical shift imaging grid is placed with a central voxel in the mid-ventricular septum and rotated to maximize coverage of the septal myocardium. The sequence uses the ultrashort echo time (UTE) approach to minimize T_2 effects and first-order phase artefacts. Five nuclear Overhauser effect (nOe) pulses (2.5 ms, 222.2 V separated by 80.5 ms) are used to increase signal to noise. Total acquisition time is approximately 10 minutes.

At the Oxford site, a 11 cm transmit/receive loop is used. A depth resolved surface coil spectroscopy (DRESS) sequence is used for localization of cardiac signal. The slice selective slab (20 mm thick) is positioned to maximize cardiac signal, while keeping parallel to the chest wall to minimize signal contamination. Total acquisition time is approximately 3.5 minutes.

Analysis

^{31}P-MRS analysis is performed in a Central Core Lab at the Oxford Centre for Clinical Magnetic Resonance Research. The spectrum from a mid-ventricular septal voxel (CSI acquisition), or from the DRESS slab, is fitted using a custom implementation of AMARES (the Advanced Method for Accurate, Robust, and Efficient Spectral fitting) in the “OXSA” semi-automated spectroscopy post-processing pipeline. Fitting uses prior knowledge specifying 11 Lorentzian peaks (α-, β-, γ-ATP multiplet components, PCr, phosphodiesterase (PDE), and 2 x 2,3-diphosphoglycerate (DPG)) and

fixed amplitude ratios and scalar couplings for the multiplets. The fitted amplitudes are then corrected for blood contamination by subtracting 30% of the average of the two 2,3-DPG signals from each of the ATP amplitudes. The remaining PCr and ATP signals are corrected for the effects of partial saturation using the flip angle at the centre of the voxel (calculated using Biot-Savart law using information about coil loading and position), assuming no motion effects and with literature T_1 values.

Farrant J, *et al. Heart* 2023; 109:1175–1182. doi: 10.1136/heartjnl-2022-322271

Supplemental statistical considerations

Sample size calculations for the mechanistic outcomes

Extracellular mass

In the pilot study, the SD of within-patient differences in myocardial extracellular mass from baseline in the trientine group was 4.2g and in the observational control group was 3.4g. Using a conservative SD of within-patient differences from baseline of 4.5g in both groups, 64 patients per group provides 87% power to detect a minimum difference, between groups, of 2.5g in terms of change in myocardial extracellular mass from baseline following 52 weeks of treatment.

Cellular mass

In the pilot study, the SD of within-patient differences in cellular mass from baseline in the trientine group was 4.9g and in the observational control group was 4.1g. Using a conservative SD of within-patient differences from baseline of 5.0g in both groups, 64 patients per group provides 80% power to detect a minimum difference, between groups, of 2.5g in terms of change in cellular mass from baseline following 52 weeks of treatment.

PCr/ATP ratio

In pilot data from HCM patients undergoing baseline and follow-up ^{31}P MRS (mean interval 6 ± 2 years), using a similar protocol to that being used here, the SD of within-patient differences in PCr/ATP ratio was 0.41. Using a conservative SD of within-patient differences from baseline of 0.55, 31 patients per group are required to detect an absolute minimum difference, between trientine and placebo groups, of 0.40 in terms of absolute change in PCr/ATP ratio from baseline following 52 weeks of treatment (80% power, 5% significance level, 2-sided). This effect size is in keeping with that seen in other studies.[5] To allow for treatment discontinuation in 25%, this is inflated to 42 per group i.e. total subgroup size 84.

Supplemental Table 1. Protocol modifications and rationale for change

**Amendments from Protocol V3.0 to Protocol V4.0**	
**Protocol section**	**Summary of changes**
Study population	Upper age limit increased from 70 to 75 years to aid recruitment.
Statistical considerations	The original sample size was inflated from 128 patients to 172 patients to allow for an anticipated treatment discontinuation rate of 25%. However, trial retention was found to be better than expected, therefore the treatment discontinuation rate was reduced to 15% i.e., total study n = 152.
**Amendments from Protocol V2.0 to Protocol V3.0**	
**Protocol section**	**Summary of changes**
Front page	Added ISRCTN number
Safety outcome measures	Removal of 'treatment emergent' from the safety outcome measures.
Participant identification and screening	Included HCM patient groups and meetings as a potential participant identification location.
Participant identification and screening	Added that Participant Identification Centres (PICs) may be used where necessary.
Table 1. Visit Schedule	Change to pregnancy test wording to allow sites to do a serum pregnancy test if timely urine testing is not available.
Pregnancy assessments	Change to pregnancy test wording to allow sites to do a serum pregnancy test if timely urine testing is not available.
Storage and shelf life	The storage conditions have changed in the SmPC to remove the need for refrigeration after opening the IMP. Wording has therefore been amended to reflect the new storage conditions.

Farrant J, *et al. Heart* 2023; 109:1175–1182. doi: 10.1136/heartjnl-2022-322271

Responsibilities - Investigator	New central safety team email account added.
**Amendments from Protocol V1.0 to Protocol V2.0**	
**Protocol section**	**Summary of changes**
Front page	Added CTA and REC reference numbers
Participant identification and screening	Wording amended to make it clear that only health care professionals can make the initial approach to the patient and not the research team.
Unblinding	Wording amended to make it clear that in the case of an emergency the decision to unblind a participant resides with the study investigators and the pharmacist should reveal the treatment allocation without approval from the CI and LCTC in this event.
Time period for safety reporting	Wording amended to state that serious adverse events or reactions will be reported from the time of informed consent, instead of from randomisation.
Flowchart for reporting requirements of adverse events	Wording amended to state that serious adverse events or reactions will be reported from the time of informed consent, instead of from randomisation.
Funding and Support in Kind	Funder reference number corrected.
Throughout	Removal of references to ICH GCP throughout protocol and replaced with Principles of GCP to ensure consistency.

Farrant J, *et al. Heart* 2023; 109:1175–1182. doi: 10.1136/heartjnl-2022-322271

Supplemental Table 2. Comparison of TEMPEST and other randomised controlled trials in HCM

Trial	Patients (n)	Design	Key inclusion criteria	Primary outcome measure
CHANCE[6]	24	1:1 candesartan (32 mg daily) : placebo	Age $\geq$ 18 years<br>LV wall thickness > 15 mm<br>LVEF $\geq$ 60%<br>Sinus rhythm<br>LVOT gradient < 30 mmHg	Primary outcome not stated.<br>Measurements included LV mass and exercise tolerance.
METAL-HCM[5]	46	1:1 perhexiline (100 mg daily) : placebo	Age 18 to 80 years<br>LV wall thickness ≥ 15 mm<br>Sinus rhythm<br>LVOT gradient < 30 mmHg<br>Exertional symptoms<br>Peak VO_2 < 75% predicted	Peak VO_2
INHERIT[7]	133	1:1 losartan (100 mg daily) : placebo	Age $\geq$ 18 years<br>LV wall thickness ≥ 15 mm or ≥ 13 mm if family history of HCM<br>LVEF $\geq$ 50%	LVMi

Farrant J, *et al. Heart* 2023; 109:1175–1182. doi: 10.1136/heartjnl-2022-322271

			Sinus rhythm<br>Any LVOT gradient	
Ho et al[8]	38	1:1 diltiazem (titrated to 360 mg daily or 5 mg/kg/day) : placebo	Age $\geq$ 5 years old<br>Normal LV wall thickness (≤ 12 mm in adults or z-score ≤ 3 in children)<br>Pathogenic or likely pathogenic HCM sarcomeric variant	Global Doppler diastolic (E′) velocity. (Changed prior to analysis to "a pilot effort to explore a broad range of imaging and biomarker features")
Maron et al[9]	53	1:1 spironolactone (50 mg daily) : placebo	Age 18 to 55 years<br>"Definitive HCM diagnosis"<br>Any LVOT gradient	Serum markers of collagen turnover (PINP, PIIINP, ICTP)
HALT-HCM[10]	42	2:1 N-acetylcysteine (2400 mg daily) : placebo	Age $\geq$ 18 years<br>LV wall thickness ≥ 15 mm<br>Preserved LV systolic function<br>Any LVOT gradient	Feasibility assessment including recruitment, retention, compliance, side effects, LV septal thickness.
RESTYLE-HCM[11]	80	1:1 ranolazine (2000 mg daily) : placebo	Age > 18 years<br>LV wall thickness ≥ 15 mm<br>Sinus rhythm	Peak VO_2

			LVOT gradient < 30 mmHg<br>NYHA II to III<br>Peak VO_2 < 75% predicted	
MAVERICK-HCM[12]	59	1:1:1 mavacamten (titrated to plasma level of 200 ng/ml) : mavacamten (titrated to plasma level of 500 ng/ml) : placebo	Age $\geq$ 18 years<br>LV wall thickness ≥ 15 mm or ≥ 13 mm if family history of HCM<br>LVEF $\geq$ 55%<br>LVOT gradient $\leq$ 30 mmHg<br>NYHA II to III<br>NTproBNP > 300 pg/ml	Safety and tolerability
EXPLORER-HCM[13]	251	1:1 mavacamten (starting dose 5mg daily, titrated according to LVOT gradient and plasma concentration) : placebo	Age $\geq$ 18 years<br>LV wall thickness ≥ 15 mm or ≥ 13 mm if family history of HCM<br>LVEF $\geq$ 55%<br>LVOT gradient $\geq$ 50 mmHg<br>NYHA II to III	Composite of:<br>• 1.5 mL/kg/min increase in peak VO_2 and $\geq$ 1 NYHA class reduction; or<br>• $\geq$ 3·0 mL/kg/min increase in peak VO_2 and no worsening of NYHA class

Farrant J, *et al. Heart* 2023; 109:1175–1182. doi: 10.1136/heartjnl-2022-322271

VANISH[14]	178	Active run in followed by 1:1 valsartan (adults: 320 mg daily; children < 18 years weighing ≥ 35 kg: 160 mg daily; children <18 years weighing <35 kg: 80 mg daily) : placebo	Age 8 to 45 years<br>LV wall thickness 12 to 25 mm<br>LVEF ≥ 55%<br>LVOT gradient ≤ 30 mmHg<br>NYHA I to II<br>Pathogenic or likely pathogenic HCM sarcomeric variant	Composite z-score, averaged from nine individual z-scores for change in the following:<br>• BSA-indexed LV mass<br>• BSA-indexed LA volume<br>• BSA-indexed LVEDV<br>• BSA-indexed LVESV<br>• BSA-adjusted maximal LV wall thickness<br>• Age-adjusted tissue Doppler diastolic (E′) velocity<br>• Age-adjusted tissue Doppler systolic (S′) velocity<br>• High-sensitivity troponin T<br>• NTproBNP
VALOR-HCM[15]	112	1:1 mavacamten (starting dose 5mg daily, titrated according to	Age ≥ 18 years<br>LV septal thickness ≥ 15 mm or ≥	Composite of:<br>• Eligible for septal reduction

		LVOT gradient and LVEF) : placebo	13 mm if family history of HCM<br>LVEF ≥ 60%<br>LVOT gradient ≥ 50 mmHg at rest or with provocation (Valsalva or exercise)<br>NYHA III to IV or II and exertional syncope/near syncope<br>Referred for septal reduction therapy and actively considering scheduling the procedure	therapy (2011 guidelines); or<br>• Patient decision to proceed with septal reduction therapy after 16 weeks of treatment
REDWOOD-HCM[16]	95	Cohort 1: 1:1 aficamten (5-15 mg) : placebo<br>Cohort 2: 1:1 aficamten (10-30 mg) : placebo<br>(The study also includes Cohorts 3 and 4, but they are not RCTs)	Age 18 to 85 years<br>LV wall thickness ≥ 15 mm or ≥ 13 mm if family history of HCM<br>LVEF ≥ 60%<br>Resting LVOT gradient ≥ 50 mmHg or resting LVOT gradient ≥ 30 and < 50 mmHg with post-Valsalva	Patient incidence of reported adverse events

			gradient ≥ 50 mmHg<br>NYHA II to III	
RESOLVE-HCM[17]	60	1:1 perhexiline (starting dose 100 mg daily, titrated according to plasma concentration) : placebo	Age ≥ 18 years<br>Interventricular septal thickness ≥15 mm<br>LVEF ≥ 55%<br>Any LVOT gradient<br>NYHA II to III and/or CCS II to III and treatment with B-blockers, and/or non-dihydropyridine calcium antagonists and/or disopyramide<br>NT-proBNP >125 pg/ml	Interventricular septal thickness
NCT05174416[18]	81	1:1 mavacamten (dose not stated) : placebo	Age ≥ 18 years<br>"Diagnosed with obstructive HCM"<br>LVEF ≥ 55%	Valsalva LVOT gradient
SEQUOIA-HCM[19]	270	1:1 aficamten (5 to 20 mg daily, titrated according to	Age 18 to 85 years<br>LV wall thickness ≥ 15 mm or ≥ 13	Peak VO_2

Farrant J, *et al. Heart* 2023; 109:1175–1182. doi: 10.1136/heartjnl-2022-322271

		echocardiography assessment) : placebo	mm if family history of HCM<br>LVEF > 60%<br>Resting LVOT gradient > 30 mmHg and post-Valsalva gradient > 50 mmHg<br>NYHA II to III<br>Respiratory exchange ratio ≥ 1.05 and peak VO_2 < 80% predicted	
TEMPEST	152	1:1 trientine (800mg daily) : placebo	Age 18 to 75 years<br>LV wall thickness ≥ 15 mm<br>LVEF > 50%<br>Any LVOT gradient<br>NYHA I to III	LVMi

BSA = body surface area; CCS = Canadian Cardiovascular Society; ICTP = C-terminal telopeptide; LA = left atrium; LVEDV = left ventricular end diastolic volume; LVESV = left ventricular end systolic volume; LVMi = body surface area-indexed left ventricular mass; LVOT = Left

Farrant J, *et al. Heart* 2023; 109:1175–1182. doi: 10.1136/heartjnl-2022-322271

ventricular outflow tract; NT proBNP = N-terminal-pro B-type natriuretic peptide; PINP = procollagen type I N-terminal propeptide; PIIINP = procollagen type III N-terminal propeptide; VO_2 = oxygen consumption. Other abbreviations as per main manuscript Table 1.

Farrant J, *et al. Heart* 2023; 109:1175–1182. doi: 10.1136/heartjnl-2022-322271

References

1. Schulz-Menger J, Bluemke DA, Bremerich J*, et al.* Standardized image interpretation and post-processing in cardiovascular magnetic resonance - 2020 update. *Journal of Cardiovascular Magnetic Resonance* 2020;**22**:19. doi: 10.1186/s12968-020-00610-6

2. Nayyar D, Nguyen T, Pathan F*, et al.* Cardiac magnetic resonance derived left atrial strain after ST-elevation myocardial infarction: an independent prognostic indicator. *Cardiovascular Diagnosis and Therapy* 2021;**11**:383-393. doi: 10.21037/cdt-20-879

3. Fröjdh F, Fridman Y, Bering P*, et al.* Extracellular Volume and Global Longitudinal Strain Both Associate With Outcomes But Correlate Minimally. *JACC Cardiovasc Imaging* 2020;**13**:2343-2354. doi: 10.1016/j.jcmg.2020.04.026

4. Messroghli DR, Moon JC, Ferreira VM*, et al.* Clinical recommendations for cardiovascular magnetic resonance mapping of T1, T2, T2* and extracellular volume: A consensus statement by the Society for Cardiovascular Magnetic Resonance (SCMR) endorsed by the European Association for Cardiovascular Imaging (EACVI). *J Cardiovasc Magn Reson* 2017;**19**:75. doi: 10.1186/s12968-017-0389-8

5. Abozguia K, Elliott P, McKenna W*, et al.* Metabolic Modulator Perhexiline Corrects Energy Deficiency and Improves Exercise Capacity in Symptomatic Hypertrophic Cardiomyopathy. *Circulation* 2010;**122**:1562-1569. doi: 10.1161/CIRCULATIONAHA.109.934059

6. Penicka M, Gregor P, Kerekes R*, et al.* The effects of candesartan on left ventricular hypertrophy and function in nonobstructive hypertrophic cardiomyopathy: a pilot, randomized study. *J Mol Diagn* 2009;**11**:35-41. doi: 10.2353/jmoldx.2009.080082

7. Axelsson A, Iversen K, Vejlstrup N*, et al.* Efficacy and safety of the angiotensin II receptor blocker losartan for hypertrophic cardiomyopathy: the INHERIT randomised, double-blind, placebo-controlled trial. *Lancet Diabetes Endocrinol* 2015;**3**:123-131. doi: 10.1016/s2213-8587(14)70241-4

8. Ho CY, Lakdawala NK, Cirino AL*, et al.* Diltiazem treatment for pre-clinical hypertrophic cardiomyopathy sarcomere mutation carriers: a pilot randomized trial to modify disease expression. *JACC Heart Fail* 2015;**3**:180-188. doi: 10.1016/j.jchf.2014.08.003

9. Maron MS, Chan RH, Kapur NK*, et al.* Effect of Spironolactone on Myocardial Fibrosis and Other Clinical Variables in Patients with Hypertrophic Cardiomyopathy. *The American Journal of Medicine* 2018;**131**:837-841. doi: 10.1016/j.amjmed.2018.02.025

10. Marian AJ, Tan Y, Li L*, et al.* Hypertrophy Regression With N-Acetylcysteine in Hypertrophic Cardiomyopathy (HALT-HCM): A Randomized, Placebo-Controlled, Double-Blind Pilot Study. *Circ Res* 2018;**122**:1109-1118. doi: 10.1161/CIRCRESAHA.117.312647

11. Olivotto I, Camici PG, Merlini PA*, et al.* Efficacy of Ranolazine in Patients With Symptomatic Hypertrophic Cardiomyopathy: The RESTYLE-HCM Randomized, Double-Blind, Placebo-Controlled Study. *Circ Heart Fail* 2018;**11**:e004124. doi: 10.1161/CIRCHEARTFAILURE.117.004124

12. Ho CY, Mealiffe ME, Bach RG*, et al.* Evaluation of Mavacamten in Symptomatic Patients With Nonobstructive Hypertrophic Cardiomyopathy. *J Am Coll Cardiol* 2020;**75**:2649-2660. doi: 10.1016/j.jacc.2020.03.064

13. Olivotto I, Oreziak A, Barriales-Villa R*, et al.* Mavacamten for treatment of symptomatic obstructive hypertrophic cardiomyopathy (EXPLORER-HCM): a randomised, double-blind, placebo-controlled, phase 3 trial. *The Lancet* 2020;**396**:759-769. doi: 10.1016/s0140-6736(20)31792-x

14. Ho CY, Day SM, Axelsson A*, et al.* Valsartan in early-stage hypertrophic cardiomyopathy: a randomized phase 2 trial. *Nat Med* 2021;**27**:1818-1824. doi: 10.1038/s41591-021-01505-4

15. Desai MY, Owens A, Geske JB*, et al.* Myosin Inhibition in Patients With Obstructive Hypertrophic Cardiomyopathy Referred for Septal Reduction Therapy. *Journal of the American College of Cardiology* 2022;**80**:95-108. doi: 10.1016/j.jacc.2022.04.048

16. REDWOOD HCM. A Multi-Center, Randomized, Double-blind, Placebo-controlled, Dose-finding Study to Evaluate the Safety, Tolerability, Pharmacokinetics, and

Pharmacodynamics of CK-3773274 in Adults With Symptomatic Hypertrophic Cardiomyopathy. https://clinicaltrials.gov/ct2/show/NCT04219826 (26/08/2022)

17. Ananthakrishna R, Lee SL, Foote J*, et al.* Randomized controlled trial of perhexiline on regression of left ventricular hypertrophy in patients with symptomatic hypertrophic cardiomyopathy (RESOLVE-HCM trial). *American Heart Journal* 2021;**240**:101-113. doi: https://doi.org/10.1016/j.ahj.2021.06.010

18. A Study to Evaluate the Efficacy and Safety of Mavacamten in Chinese Adults With Symptomatic Obstructive HCM. https://clinicaltrials.gov/ct2/show/NCT05174416 (26/08/2022)

19. SEQUIA-HCM. A Phase 3, Multi-Center, Randomized, Double-blind, Placebo-controlled Trial to Evaluate the Efficacy and Safety of CK-3773274 in Adults With Symptomatic Hypertrophic Cardiomyopathy and Left Ventricular Outflow Tract Obstruction https://www.clinicaltrials.gov/ct2/show/NCT05186818 (12/10/2022)
